# I want my money back

**DOI:** 10.1017/S1478951526102259

**Published:** 2026-04-13

**Authors:** Zhaohui Su

**Affiliations:** 1 School of Public Health, Southeast University, Nanjing, China; 2 Key Laboratory of Environmental Medicine Engineering, Ministry of Education, School of Public Health, Southeast University, Nanjing, China

Time is a great teacher,
The one that kills all its pupils.

The lessons often start with
“Everything is beautiful,”
Then they promise
“After rain come rainbows,”
They continue with
“All pains are growing pains,”
And at some point, they end with
“The world is far too tangled for lessons at all.”

Time is a great teacher,
Yet its lessons are far from ideal.
Between lessons “too much, too early,”
And lessons “too little, too late,”
I want my money back.

Life is a great teacher,
The one that buries all its pupils.

The lessons first disguised as conclusions –
Rules of the game written only by survivors;
Then they pose as questions –
Why not choose the path to an easy continuation?
They grow into threats –
Daylight can become a nightmare by the will of the powerful;
And finally reveal the ultimate challenge –
What if everything means little from the get-go?

Time is a great teacher,
Yet its lessons are far from ideal.
Between lessons “too broad, too philosophical,”
And lessons “too narrow, too cynical,”
I want my money back.

## Data Availability

Data are available upon reasonable request.

